# SEOM clinical guideline for treatment of kidney cancer (2017)

**DOI:** 10.1007/s12094-017-1765-4

**Published:** 2017-11-13

**Authors:** E. Gallardo, M. J. Méndez-Vidal, J. L. Pérez-Gracia, J. M. Sepúlveda-Sánchez, M. Campayo, I. Chirivella-González, X. García-del-Muro, A. González-del-Alba, E. Grande, C. Suárez

**Affiliations:** 1 Medical Oncology Department, Parc Taulí Hospital Universitari, Institut d’Investigació i Innovació Parc Taulí I3PT, Universitat Autònoma de Barcelona, Parc Taulí, 1, 08208 Sabadell, Spain; 2 Medical Oncology Department, Maimonides Institute of Biomedical Research (IMIBIC), Reina Sofía Hospital, University of Córdoba, Córdoba, Spain; 3 Medical Oncology Department, Clinica Universidad de Navarra, Pamplona, Spain; 4 Medical Oncology Department, Hospital Universitario 12 de Octubre, Madrid, Spain; 5 Medical Oncology Department, Hospital Universitari Mutua Terrassa, Terrassa, Spain; 6 Medical Oncology Department, Hospital Clínico, Universidad de Valencia, Valencia, Spain; 7 Medical Oncology Department, Institut Catala d’Oncologia, Idibell, Universitat de Barcelona, Barcelona, L’Hospitalet de Llobregat Spain; 8 Oncology Department, Hospital Universitario Son Espases, Palma De Mallorca, Spain; 9 Medical Oncology Department, Hospital Universitario Ramón y Cajal, Madrid, Spain; 10 Medical Oncology Department, Vall d’Hebron University Hospital and Institute of Oncology, Universitat Autònoma de Barcelona, Barcelona, Spain

**Keywords:** Kidney cancer, Systemic therapy, Molecular pathology

## Abstract

The goal of this article is to provide recommendations about the management of kidney cancer. Based on pathologic and molecular features, several kidney cancer variants were described. Nephron-sparing techniques are the gold standard of localized disease. After a randomized trial, sunitinib could be considered in adjuvant treatment in high-risk patients. Patients with advanced disease constitute a heterogeneous population. Prognostic classification should be considered. Both sunitinib and pazopanib are the standard options for first-line systemic therapy in advanced renal cell carcinoma. Based on the results of two randomized trials, both nivolumab and cabozantinib should be considered the standard for second and further lines of therapy. Response evaluation for present therapies is a challenge.

## Introduction

According to GLOBOCAN 2012, 338,000 new kidney cancer cases were diagnosed in the world, what implies around 5% of men and 3% of women, with an age-standardized rate (ASR) of 8.5 cases per 100,000-person-year [1]. This means the 8th most frequent tumor among men and the 14th among women. In addition, a number of 144,000 deaths due to kidney cancer occurred worldwide. In Spain, the estimated incidence in 2015 was 3590 cases, with an ASR of 15.8 cases per 100,000-person-year [2].

Comparing to former statistics, kidney cancer incidence is progressively stabilizing or decreasing. Differences have been observed among geographic areas, with the highest incidence rates in developed regions. Most renal cancers (75%) are diagnosed over the age of 60. No differences among races seem apparent.

There are several well-established epidemiologic risk factors: smoking, obesity, hypertension, and familial cancer syndromes [3]. Approximately, 2–3% of kidney cancer cases are related to a hereditary autosomal dominant syndrome, the most frequent of whom is von Hippel–Lindau syndrome associated with clear-cell renal cell carcinoma. Several other factors have been related, such as end-stage renal disease, parity in women, and toxic exposure like trichloroethylene.

## Methodology

The SEOM guidelines have been developed with the consensus of ten genitourinary cancer oncologists from SEOM (Spanish Society of Medical Oncology) and SOGUG (Spanish Oncology Genitourinary Group). To assign a level of levels of evidence and grades of recommendation, we have used Table 1 [4]. Statements without grading were considered justified standard clinical practice by the SEOM/SOGUG faculty and experts.

**Table 1:** Levels of evidence/grades of recommendation

Levels of evidence
I Evidence from at least one large randomized, controlled trial of good methodological quality (low potential for bias) or meta-analyses of well-conducted randomized trials without heterogeneity
II Small randomized trials or large randomized trials with a suspicion of bias (lower methodological quality) or meta-analyses of such trials or of trials with demonstrated heterogeneity
III Prospective cohort studies
IV Retrospective cohort studies or case–control studies
V Studies without control group, case reports, experts opinions
Grades of recommendation
A Strong evidence for efficacy with a substantial clinical benefit, strongly recommended
B Strong or moderate evidence for efficacy but with a limited clinical benefit, generally recommended
C Insufficient evidence for efficacy or benefit does not outweigh the risk or the disadvantages; optional
D Moderate evidence against efficacy or for adverse outcome, generally not recommended
E Strong evidence against efficacy or for adverse outcome, never recommended

## Diagnosis and staging

More than 50% of renal cell carcinomas (RCC) are detected incidentally. The classic triad of flank pain, visible haematuria, and palpable abdominal mass is rare (6–10%) and correlates with aggressive histology and advanced disease. Paraneoplastic syndromes are found in approximately 30% of patients with symptomatic RCC. Some symptomatic patients present with symptoms caused by metastatic disease, such as bone pain or persistent cough.

Abdominal computed tomography (CT) scan represents the gold standard in the staging of RCC. Enhancement in renal masses is determined by comparing Hounsfield units (HU) before and after contrast administration; a change of 15 or more HU suggests malignancy [5]. Abdominal CT provides information for staging: function and morphology of the contralateral kidney; primary tumor extension; venous involvement; locoregional lymph nodes status; adrenal glands; and other solid organs involvement [6]. Contrast-enhanced CT angiography is useful in selected cases for detailed information on renal vascular supply.

Abdominal magnetic resonance imaging (MRI) is not performed routinely, but may provide additional information on venous involvement [7]. MRI is indicated in patients allergic to intravenous CT contrast medium and in pregnancy without renal failure [8]. Despite a high accuracy of both CT and MRI in RCC diagnosis, these tests are not able to reliably distinguish oncocytoma and fat-free angiomyolipoma from RCC [9].

For evaluation of advanced disease, chest CT is accurate for chest staging [10]. Since most bone metastases are symptomatic at diagnosis, routine bone imaging is not generally indicated [11]. However, bone scan and brain CT or MRI should be used if specific clinical or laboratory signs and symptoms are present. In patients with impaired renal function, an isotope renogram and total renal function evaluation should be considered. In general, positron-emission tomography (PET) is not recommended [12].

The staging of RCC is done according to the eighth TNM classification system (2017) that is used for all histologic variants of renal carcinoma. This system is supported by both the American Joint Committee on Cancer (AJCC) and the International Union for Cancer Control (UICC) [13]. The TNM system is shown in Table 2. Stage grouping for RCC based on AJCC TNM 2017 is shown in Table 3.

**Table 2:** Kidney cancer TNM staging AJCC UICC 2017

*Primary tumor (T)*	
T category	T criteria
TX	Primary tumor cannot be assessed
T0	No evidence of primary tumor
T1	Tumor ≤ 7 cm in greatest dimension, limited to the kidney
T1a	Tumor ≤ 4 cm in greatest dimension, limited to the kidney
T1b	Tumor > 4 cm but ≤ 7 cm in greatest dimension, limited to the kidney
T2	Tumor > 7 cm in greatest dimension, limited to the kidney
T2a	Tumor > 7 cm but ≤ 10 cm in greatest dimension, limited to the kidney
T2b	Tumor > 10 cm, limited to the kidney
T3	Tumor extends into major veins or perinephric tissues, but not into the ipsilateral adrenal gland and not beyond Gerota’s fascia
T3a	Tumor extends into the renal vein or its segmental branches, or invades the pelvicalyceal system, or invades perirenal and/or renal sinus fat but not beyond Gerota’s fascia
T3b	Tumor extends into the vena cava below the diaphragm
T3c	Tumor extends into the vena cava above the diaphragm or invades the wall of the vena cava
T4	Tumor invades beyond Gerota’s fascia (including contiguous extension into the ipsilateral adrenal gland)
*Regional lymph nodes (N)*	
N category	N criteria
Nx	Regional lymph nodes cannot be assessed
N0	No regional lymph node metastasis
N1	Metastasis in regional lymph node(s)
*Distant metastasis (M)*	
M category	M criteria
M0	No distant metastasis
M1	Distant metastasis

**Table 3:** Stage grouping for RCC based on AJCC TNM 2017

Stage	T	N	M
I	T1	N0	M0
II	T2	N0	M0
III	T1 or 2	N1	M0
	T3	Any N	M0
IV	T4	Any N	M0
	Any T	Any N	M1

## Recommendations

- Abdominal CT scan is the gold standard for staging of RCC and provides information on primary, regional, and metastatic involvement. Level of evidence: III. Grade of recommendation: A.
- Abdominal MRI is an alternative in several circumstances. Chest CT is recommended for thorax staging. Bone scan and brain studies are not routinely recommended. Level of evidence: III. Grade of recommendation: B.

## Pathological and molecular classification

Among kidney cancers, completely different entities can be found from both the histology and molecular perspective. It is estimated that around 85% of kidney tumors are RCC being the clear cell (ccRCC) histology the most frequent one, accounting for up to 75–80% of all RCC [14]. The genomic study defined by the Cancer Genome Atlas (TCGA) of more than 400 ccRCC samples revealed the importance of mutations of genes related to angiogenesis, mainly the *von Hippel Lindau (VHL) gene*, together with mutations altering the chromatin remodelling complexes (*PBRM1, ARID1A,* and *SMARCA4*) and other epigenetic regulators such as *SETD2* and *BAP1*. It was also observed that 28% of the samples are harbouring mutations affecting the *PI3K/Akt* pathway that directly affect metabolic intracellular routes [15]. Among patients with non-clear cell histology (nccRCC), papillary RCC (pRCC) is the most frequent one and comprises around 10–15% of RCC cases. Papillary tumors include two main subtypes (type I and type II), which differ in their molecular drivers and prognosis. Type I pRCC is mostly associated with mutations in the *MET* oncogene and exerts a more favorable prognosis, while type II patients use to harbour aberrations in the Krebs cycle gene *fumarate hydratase* (*FH*) that confer a very poor prognosis in most cases [16]. Sarcomatoid features are present in 1–8% of RCC tumors mostly seen in patients with predominant clear cells areas. Other non-ccRCC subtypes include chromophobe (chRCC) tumors with an incidence rate of ~ 5%, collecting duct tumors (< 1%) and more rare cases like Xp11 translocation (tRCC) or medullary subtypes that exert a poor clinical outcome despite of systemic treatment [17]. In addition, there are around 4–5% of tumors that remain unclassified.

The distinct histology tumor subtypes are conditioning different sensitivity to the broad range of systemic available therapies for metastatic RCC (mRCC). Beyond the pathological subtype, the TCGA of ccRCC identified four stable subsets in both mRNA (m1–m4) and miRNA (mi1–mi4) expression data sets [15]. What it seems to be more important is that there could be a relationship between these molecular subgroups and the sensitivity to tyrosine kinase inhibitors (TKI). In this regard, sunitinib might not work in those patients with ccrcc-1 and -4 subgroups (c-myc and immune-like profiles, respectively) that it does in ccrcc-2 and -3 (normal-like, and classical subtypes, respectively) as shown in a retrospective analysis conducted in 53 patients with metastatic ccRCC [18]. More recently, it has been shown that molecular profiling could also help to identify not only patients that are sensitive to be treated with antiangiogenics but also those that are most likely to respond to novel immuno-oncology agents [19].

## Local and locoregional disease

Surgery is the treatment of choice for localized renal cell cancer. Partial nephrectomy (nephron-sparing surgery) is indicated in tumors smaller than 7 cm if technically feasible. This approach is associated with better long-term preservation of renal function and similar oncological outcomes than radical surgery. However, this procedure is not always technically feasible, mainly due to anatomical or surgical factors. In these cases, laparoscopic radical nephrectomy is an alternative. Partial nephrectomy is also the preferred approach for patients with bilateral tumors or a single functional kidney. Radical nephrectomy is indicated in T2-4 tumors. Laparoscopic approach is preferred to open radical nephrectomy in T2 and selected T3a tumors, because it is associated with less surgical-related complications. In T3b and T4 tumors, open radical nephrectomy is the approach of choice. When a tumor thrombus is present, it has to be completely excised. Extended lymphadenectomy and adrenalectomy have not shown added survival benefit and should not be routinely performed unless there is evidence of involvement.

Radiofrequency and cryotherapy are local ablative techniques in development that constitute a therapeutic alternative in elderly or high-risk patients with small renal cancers, as well as in hereditary RCC syndromes, bilateral tumors, or single functional kidney. Initial active surveillance is also an acceptable alternative in elderly or high-risk patients with small renal masses. Patients should be followed with repeated abdominal imaging and surgery performed in those cases that show clinical progression during the follow-up.

Several different classifications have been proposed to assess the risk of recurrence in patients with localized renal cell cancer treated with nephrectomy [20]. Regarding the role of systemic therapies in patients with high risk of relapse a recent study has shown a significant improvement in disease-free survival (DFS) in patients who received adjuvant sunitinib for 1 year [21]. This benefit seems to be especially apparent in the group of patients with higher risk features. Unfortunately, mature overall survival (OS) data are not available yet. Moreover, toxicity of sunitinib was considerable in this population. On the other hand, another study comparing 1-year treatment with sunitinib, sorafenib or placebo showed no differences in terms of DFS between arms [22]. However, differences in population prognostic features and dose intensity of therapy between both studies are remarkable. At this moment, 1-year adjuvant therapy with sunitinib could be a non-approved individualized alternative to consider in selected high-risk patients.

Neoadjuvant therapy for localized renal cell cancer has been studied in several small clinical trials. Their results suggest that this approach is feasible, and might be especially useful in large unresectable masses, high-level venous tumor thrombus involvement, and patients with large masses and imperative indications for nephron sparing surgery. Nevertheless, at present, this approach still remains investigational.

## Recommendations

- Partial nephrectomy is recommended in T1 tumors, if technically feasible, as well as in bilateral tumors or a single functional kidney. Radical nephrectomy is recommended in T2-4 tumors. Level of evidence: III. Grade of recommendation: A.
- Adjuvant therapy with sunitinib over 1 year after nephrectomy could be an option to consider individually in patients with high-risk features. However, there is still insufficient evidence to recommend this therapy routinely in clinical practice. Level of evidence: II. Grade of recommendation: C.

## Advanced disease

### Prognostic classification

A number of tumor and host characteristics have been found useful in predicting the risk of death from metastatic kidney cancer. The Memorial Sloan-Kettering Cancer Center (MSKCC) criteria defined the pretreatment features that predicted survival in 463 patients with mRCC treated with interferon alfa (IFNα) in clinical trials and have been widely used [23]. The MSKCC risk system classifies patients with mRCC into three categories: poor, intermediate, and favorable risks. Multivariate analysis showed five variables as independent adverse prognostic factors: Karnofsky performance status (KPS) less than 80%, interval from diagnosis to treatment of less than 1 year, serum hemoglobin level less than the lower limit of normality (LLN), serum lactate dehydrogenase (LDH) greater than 1.5 times the upper limit of normality (ULN) and corrected serum calcium greater than the ULN. Those patients with none of these factors were classified as low risk (good prognosis), those with 1 or 2 factors were considered intermediate risk, and patients with 3 or more factors were considered poor risk.

Trials with patients treated with contemporary VEGF-targeted therapies have been analyzed to outline a newer prognosis classification. The International Metastatic Database Consortium (IMDC) retrospectively assessed 645 patients with mRCC treated with sorafenib, sunitinib or bevacizumab-IFNα and identified six variables to classify cases into favorable, intermediate and poor prognosis groups [24]: KPS less than 80%, time from nephrectomy less than 1 year, hemoglobin less than LLN, serum corrected calcium greater than ULN, platelets greater than ULN and absolute neutrophil count greater than ULN. Data from these patients were used to generate a similar model that can be used to predict survival in second-line therapy after progression to VEGF-targeted agents [25] and also in patients with non-clear mRCC [26]. Table 4 summarizes MSKCC (Motzer) and IMDC (Heng) risk criteria.

**Table 4:** MSKCC and IMDC risk criteria for poor overall survival

MSKCC criteria	IMDC criteria
KPS < 80	KPS < 80
Diagnosis to therapy < 1 year	Diagnosis to therapy < 1 year
Anemia	Anemia
Hypercalcemia	Hypercalcemia
Elevated lactate dehydrogenase	Thrombocytosis
	Neutrophilia

For both classifications:

0 factors: favorable risk

1–2 factors: intermediate risk

3 or more factors: poor risk

### Recommendation

- Prognostic classifications, such as MSKCC and IMDC, should be used for management of mRCC patients. Level of evidence: II. Grade of recommendation: B.

### Role of surgery

Two prospective clinical trials assessed the role of debulking or cytoreductive nephrectomy in patients with mRCC treated with IFNα. Both studies randomized patients to receive IFNα alone or nephrectomy followed by IFNα, finding a significant improvement in terms of survival favoring the nephrectomy approach [27]. However, the mechanism responsible for this beneficial effect remains unclear and patients should be carefully selected. Patients most likely to benefit from nephrectomy include those with resectable primary tumor, good performance status, adequate organ function, and no significant comorbidities or involvement of central nervous system [28]. Recommendations and level of evidence are provided in Table 5.

**Table 5:** SEOM guideline recommendations for kidney cancer

Diagnosis and staging
Abdominal CT scan is the gold standard for staging of RCC and provides information on primary, regional and metastatic involvement. Level of evidence: III. Grade of recommendation: A
Abdominal MRI is an alternative in several circumstances. Chest CT is recommended for thorax staging. Bone scan and brain studies are not routinely recommended. Level of evidence: III. Grade of recommendation: B
Local and locoregional disease
Partial nephrectomy is recommended in T1 tumors, if technically feasible, as well as in bilateral tumors or a single functional kidney. Radical nephrectomy is recommended in T2-4 tumors. Level of evidence: III. Grade of recommendation: A
Adjuvant therapy with sunitinib over 1 year after nephrectomy could be an option to consider individually in patients with high-risk features. However, there is still insufficient evidence to recommend this therapy routinely in clinical practice. Level of evidence: II. Grade of recommendation: C
Prognostic classification
Prognostic classifications, such as MSKCC and IMDC, should be used for management of mRCC patients. Level of evidence: II. Grade of recommendation: B
Surgery in advanced disease
Debulking or cytoreductive nephrectomy is the standard of care for selected mRCC patients with good or intermediate prognosis, however this procedure should be avoided in the majority of patients with poor-risk features. Level of evidence: III. Grade of recommendation: B
Metastasectomy can be considered in selected patients with limited number of metastases with long metachronous disease-free interval Level of evidence: III. Grade of recommendation: B
First-line treatment in advanced disease
In patients with good or intermediate prognosis, sunitinib and pazopanib are the most recommended options for the first-line treatment of mRCC with clear-cell histology. Level of evidence: I.,Grade of recommendation: A
For patients with poor prognosis, temsirolimus is the only option supported by a phase III trial. Level of evidence: I. Grade of recommendation: A
Sunitinib and pazopanib have also shown benefit in the treatment of poor-prognosis patients. Level of evidence: III. Grade of recommendation: B
Second-line treatment in advanced disease
Nivolumab and cabozantinib have shown increased OS in patients with advanced ccRCC previously treated with antiangiogenics, and are the recommended treatments for these patients. Level of evidence: I. Grade of recommendation: A
Decisions to use either agent may be based on the expected toxicity and on contraindications for each drug, as randomized data is lacking. Level of evidence: IV. Grade of recommendation: D
Lenvatinib in combination with everolimus has shown increased OS in patients with advanced ccRCC in a randomized phase II trial, and is another valid alternative for these patients. Level of evidence: II. Grade of recommendation: B
Axitinib and everolimus have not shown increased OS after prior antiangiogenic therapy and should not be used before the previous agents. Nevertheless they may remain acceptable options following such agents, although they have not been tested in randomized trials in this setting. Level of evidence: II. Grade of recommendation: B
Non-clear cell renal cell carcinoma
VEGFR inhibitors, such as sunitinib, are the preferred option for papillary RCC. Level of evidence: II. Grade of recommendation: B
Response evaluation and follow-up
After a definitive treatment for a localized renal cell carcinoma a follow up should be planned. Level of evidence: V. Grade of recommendation: B

The role of cytoreductive nephrectomy in patients who receive subsequent targeted therapy is currently under evaluation in three prospective trials investigating sunitinib or pazopanib with or without nephrectomy in patients with mRCC. Retrospective evidence from the IMDC with data of 1658 patients showed significantly longer OS in the group of cytoreductive nephrectomy in patients with favorable and intermediate prognosis, nevertheless in patients with poor prognosis debulking nephrectomy did not provide any benefit [29].

Metastasectomy may be considered in mRCC patients with favorable prognostic features: good performance status (PS), limited metastatic disease, prolonged time between initial diagnosis, and development of metastases and the possibility for a complete resection [30].

## Recommendations

- Debulking or cytoreductive nephrectomy is the standard of care for selected mRCC patients with good or intermediate prognosis; however, this procedure should be avoided in the majority of patients with poor-risk features. Level of evidence: III. Grade of recommendation: B.
- Metastasectomy can be considered in selected patients with limited number of metastases with long metachronous disease-free interval Level of evidence: III. Grade of recommendation: B.

### First-line systemic treatment

The current standard of care in the first-line setting focuses on the inhibition of angiogenesis. In this scenario, either sunitinib, bevacizumab plus IFNα, or pazopanib improved progression-free survival (PFS) compared with IFNα or placebo in patients with good or intermediate prognosis, with PFS of 8.5–11 months [31–34]. Although similar benefit was seen with bevacizumab plus IFNα, sunitinib and pazopanib, oral VEGFR tyrosine kinase inhibitors (TKI), have become the standard of care in this situation. Both sunitinib and pazopanib were compared in the non-inferiority phase III COMPARZ trial, which demonstrated no difference in outcomes with the two agents [35]. Nevertheless, no predictors of response to targeted therapy are available; thereby, the choice of therapy is usually based on the patient’s prognostic profile, patient and physician preference and experience, and drug efficacy and toxicity profiles.

In patients with poor-risk features, the mTOR inhibitor temsirolimus has been shown to improve OS compared with IFNα alone and represents the only option supported with level I evidence [36]. Other alternatives include sunitinib, pazopanib, and bevacizumab combined with IFNα, based on the minimal inclusion of poor-risk patients in pivotal trials and expanded-access studies of these drugs.

Immunotherapy with high-dose interleukin-2 (HD-IL2) remains a viable therapeutic option in centers with experience for patients with good prognosis mRCC clear-cell histology and low-volume disease. The full potential of checkpoint inhibitors in the front-line setting is under investigation.

## Recommendations

- In patients with good or intermediate prognosis, sunitinib and pazopanib are the most recommended options for the first-line treatment of mRCC with clear-cell histology. Level of evidence: I., Grade of recommendation: A.
- For patients with poor prognosis, temsirolimus is the only option supported by a phase III trial. Level of evidence: I. Grade of recommendation: A.
- Sunitinib and pazopanib have also shown benefit in the treatment of poor-prognosis patients. Level of evidence: III. Grade of recommendation: B.

### Second line and sequence

Current options for treatment of advanced ccRCC in second line and beyond include immunotherapy with PD-1 blockade and TKI. Nivolumab, an antibody against PD-1, and cabozantinib, an oral TKI targeting VEGFR, MET and AXL, were compared with everolimus, an mTOR inhibitor previously approved in second line of treatment of mRCC, in two different randomized phase III trial including 821 and 658 patients with mRCC patients previously treated with at least one prior antiangiogenic therapy one prior antiangiogenic therapy [37, 38] Both cabozantinib and nivolumab increased OS (figures of 21.4 and 25.0 months, respectively) and response rate (RR), while PFS was significantly better for the former and no differences were observed for the latter. However, toxicity profiles were different, with less grade 3–4 adverse events and treatment discontinuations for nivolumab compared to everolimus. On the other hand, 60% of patients treated with cabozantinib required dose reductions due to toxicity. Based on this data, both nivolumab and cabozantinib has been granted approval for this indication by regulatory agencies [IA, A].

The combination of lenvatinib, another oral TKI of VEGFR1-3, FGFR1-4, PDGFRα, RET, and KIT, and everolimus was compared in a randomized phase II study with either everolimus or lenvatinib alone in patients with mRCC treated with one previous VEGF-targeted therapy [39]. Significant differences for OS, PFS, and RR were described for lenvatinib plus everolimus compared to everolimus alone. Dose reductions due to toxicity and treatment discontinuation because of adverse events in patients allocated to lenvatinib plus everolimus were required, respectively, in 71 and 24% of cases, respectively. Based on this data, the FDA approved lenvatinib in combination with everolimus in this setting [IB, B].

No direct comparisons have been performed between any PD-1 blocking therapy and the TKI that increase OS in these patients, and no valid biomarkers exist to select the most appropriate treatment for each patient. Therefore, decisions to use these options should be guided by clinical characteristics (e.g., contraindications for immunotherapy (e.g., autoimmune diseases, organ transplant) or to TKI (e.g., uncontrollable hypertension, intolerance) and by the toxicity expected for each agent [IV, D].

Other TKI, such as axitinib [40] and sorafenib [41] and mTOR inhibitors, such as everolimus [42] have not shown increased OS after prior antiangiogenic therapy and should not be used before the previous agents. Axitinib against sorafenib, and everolimus, against placebo, demonstrated PFS benefit in phase III trials. Yet, they may remain acceptable options afterwards, although randomized trials in this setting are unavailable [IV, D].

## Recommendations

- Nivolumab and cabozantinib have shown increased OS in patients with advanced ccRCC previously treated with antiangiogenics, and are the recommended treatments for these patients. Level of evidence: I. Grade of recommendation: A.
  - Decisions to use either agent may be based on the expected toxicity and on contraindications for each drug, as randomized data is lacking. Level of evidence: IV. Grade of recommendation: D.
- Lenvatinib in combination with everolimus has shown increased OS in patients with advanced ccRCC in a randomized phase II trial, and is another valid alternative for these patients. Level of evidence: II. Grade of recommendation: B.
- Axitinib and everolimus have not shown increased OS after prior antiangiogenic therapy and should not be used before the previous agents. Nevertheless, they may remain acceptable options following such agents, although they have not been tested in randomized trials in this setting. Level of evidence: II. Grade of recommendation: B.

### Non clear-cell renal cell carcinoma

While non-clear cell histologies constitute a minority of cases of RCC, they pose a significant therapeutic challenge. Non-ccRCC are characterized by morphology, growth pattern, cell of origin, and by the histochemical and biologic bases that underlie the different types of tumors.

The general approach to treatment of non-ccRCC mirrors that for ccRCC. A meta-analysis of targeted therapy clinical trials suggests that VEGF-targeting agents may have activity in patients with both non-clear cell or clear cell histologies with sarcomatoid features [43]. Another meta-analysis comparing effectiveness and adverse effects of different systemic treatments for non-ccRCCs described that single studies showed a trend towards favoring sunitinib in terms of OS and PFS (HR 1.41, 80% confidence interval) [44]. These tumors do not respond to immunotherapy with IL-2, although dramatic responses have been reported with anti-PD-1 [45]. Besides the identification of genomic basis, results from some phase II, randomized trials are of interest for these RCC subtypes.

The ESPN [46] and ASPEN [47] studies compared sunitinib and everolimus in patients with metastatic non-ccRCC (or ccRCC with > 20% sarcomatoid features in ESPN). Primary endpoint was PFS in first-line therapy for both trials. Results were obtained from 68 and 108 patients, respectively. For both trials, although not statistically significant, sunitinib was superior overall compared with everolimus at delaying disease progression. However, it was also associated with a higher rate of severe toxicity. Interestingly, sunitinib was found to be more effective for papillary-type kidney cancers and for better prognosis patients. On the other hand, patients with chromophobe and poor-risk tumors treated with everolimus had a longer median PFS.

In summary, the evidence base concerning the treatment of this group of patients is relatively small. Although activity with VEGFR TKI or mTOR inhibitors has been observed, shorter survival times and lower response rates compared to ccRCC patients highlight continuing medical need for new treatment approaches in this patient population.

## Recommendation

- VEGFR inhibitors, such as sunitinib, are the preferred option for papillary RCC. Level of evidence: II. Grade of recommendation: B.

### Response evaluation and follow-up

For patients with advanced renal cell carcinoma on systemic treatment, response evaluation is generally performed every 2–3 months with a CT scan of the thorax and abdomen. Response Evaluation Criteria in Solid Tumors (RECIST) is still the standard method to assess drug response or resistance, although caution is needed to avoid false interpretations of progression of the disease. In this setting, other evaluation methods could better correlate treatment with TKI or immunotherapies with clinical outcome, but its use in the daily clinical practice is not available yet [48, 49].

There is no standard protocol for the follow-up after a definitive treatment for a localized renal cell carcinoma. The higher risk of recurrence following surgical resection is in the first 3 years being 1–2 years the median time to relapse. For this reason, the follow-up must be more intensive on this period. However, there is no clear recommendation about the timing and number of tests to perform. The most extended imaging test is the CT scan of the chest and abdomen. Several surveillance protocols use a risk-stratified approach and are useful to define the best follow-up strategy for each patient [50]. These protocols take many variables into account such us TNM stage, Fuhrman grade or type of local treatment (partial versus radical nephrectomy). For patients with a high or intermediate risk of relapse, a closer follow-up is recommended, especially for the first 2–3 years (CT scan every 3–6 months), while a less frequent follow-up is needed for patients with a low risk of relapse and after 3 years of surveillance. Moreover, the optimal duration of surveillance is not clear either. Due to the presence of late relapses, further follow-up after 5 years is performed in some institutions, especially for patients with intermediate or high risk.

## Recommendation

- After a definitive treatment for a localized renal cell carcinoma, a follow-up should be planned. Level of evidence: V. Grade of recommendation: B. Recommendations and level of evidence are provided in Table 5

## References

[1] Ferlay J, Soerjomataram I, Dikshit R, Eser S, Mathers C, Rebelo M (2015). Cancer incidence and mortality worldwide: sources, methods and major patterns in GLOBOCAN 2012. Int J Cancer.

[2] Galceran J, Ameijide A, Carulla M, Mateos A, Quirós JR, Rojas D (2017). Cancer incidence in Spain, 2015. Clin Transl Oncol.

[3] Chow WH, Dong LM, Devesa SS (2010). Epidemiology and risk factors for kidney cancer. Nat Rev Urol.

[4] Dykewicz CA, Centers for Disease Control and Prevention (U.S.), Infectious Diseases Society of America, American Society of Blood and Marrow Transplantation (2001). Summary of the guidelines for preventing opportunistic infections among hematopoietic stem cell transplant recipients. Clin Infect Dis.

[5] Israel GM, Bosniak MA (2008). Pitfalls in renal mass evaluation and how to avoid them. Radiographics.

[6] Sokhi HK, Mok WY, Patel U (2015). Stage T3a renal cell carcinoma: staging accuracy of CT for sinus fat, perinephric fat or renal vein invasion. Br J Radiol.

[7] Mueller-Lisse UG, Mueller-Lisse UL (2010). Imaging of advanced renal cell carcinoma. World J Urol.

[8] Putra LG, Minor TX, Bolton DM, Appu S, Dowling CR, Neerhut GJ (2009). Improved assessment of renal lesions in pregnancy with magnetic resonance imaging. Urology.

[9] Choudhary S, Rajesh A, Mayer NJ, Mulcahy KA, Haroon A (2009). Renal oncocytoma: CT features cannot reliably distinguish oncocytoma from other renal neoplasms. Clin Radiol.

[10] Lim DJ, Carter MF (1993). Computerized tomography in the preoperative staging for pulmonary metastases in patients with renal cell carcinoma. J Urol.

[11] Koga S, Tsuda S, Nishikido M, Ogawa Y, Hayashi K, Hayashi T (2001). The diagnostic value of bone scan in patients with renal cell carcinoma. J Urol.

[12] Park JW, Jo MK, Lee HM (2009). Significance of 18F-fluorodeoxyglucose positron-emission tomography/computed tomography for the postoperative surveillance of advanced renal cell carcinoma. BJU Int..

[13] Rini BI, McKiernan JM, Chang SS, Amin MB (2017). Kidney. AJCC cancer staging manual.

[14] Udager AM, Mehra R (2016). Morphologic, molecular, and taxonomic evolution of renal cell carcinoma: a conceptual perspective with emphasis on updates to the 2016 World Health Organization Classification. Arch Pathol Lab Med.

[15] Cancer Genome Atlas Research Network (2013). Comprehensive molecular characterization of clear cell renal cell carcinoma. Nature.

[16] Linehan WM, Spellman PT, Ricketts CJ, Creighton CJ, Fei SS, Davis C, Cancer Genome Atlas Research Network (2016). Comprehensive molecular characterization of papillary renal-cell carcinoma. N Engl J Med.

[17] Davis CF, Ricketts CJ, Wang M, Yang L, Cherniack AD, Shen H (2014). The somatic genomic landscape of chromophobe renal cell carcinoma. Cancer Cell.

[18] Beuselinck B, Job S, Becht E, Karadimou A, Verkarre V, Couchy G (2015). Molecular subtypes of clear cell renal cell carcinoma are associated with sunitinib response in the metastatic setting. Clin Cancer Res.

[19] McDermott D, Huseni M, Rini B, Motzer R, Atkins M, Escudier B, et al. Molecular correlates of differential response to Atezolizumab ± Bevacizumab vs sunitinib in a phase II study in untreated metastatic renal cell carcinoma (RCC) patients. Presented as an oral abstract communication at the AACR 2017 meeting. Washington. Abstract CT081.

[20] Zisman A, Pantuck AJ, Wieder J, Chao DH, Dorey F, Said JW (2002). Risk group assessment and clinical outcome algorithm to predict the natural history of patients with surgically resected renal cell carcinoma. J Clin Oncol.

[21] Ravaud A, Motzer RJ, Pandha HS, George DJ, Pantuck AJ, Patel A (2016). Adjuvant sunitinib in high-risk renal-cell carcinoma after nephrectomy. N Engl J Med.

[22] Haas NB, Manola J, Uzzo RG, Flaherty KT, Wood CG, Kane C (2016). Adjuvant sunitinib or sorafenib for high-risk, non-metastatic renal-cell carcinoma (ECOG-ACRIN E2805): a double-blind, placebo-controlled, randomised, phase 3 trial. Lancet.

[23] Motzer RJ, Bacik J, Murphy BA, Russo P, Mazumdar M (2002). Interferon-alfa as a comparative treatment for clinical trials of new therapies against advanced renal cell carcinoma. J Clin Oncol.

[24] Heng DY, Xie W, Regan MM, Warren MA, Golshayan AR, Sahi C (2009). Prognostic factors for overall survival in patients with metastatic renal cell carcinoma treated with vascular endothelial growth factor-targeted agents: results from a large, multicenter study. J Clin Oncol.

[25] Ko JJ, Xie W, Kroeger N, Lee JL, Rini BI, Knox JJ (2015). The international Metastatic Renal Cell Carcinoma Database Consortium model as a prognostic tool in patients with metastatic renal cell carcinoma previously treated with first-line targeted therapy: a population-based study. Lancet Oncol.

[26] Kroeger N, Xie W, Lee JL, Bjarnason GA, Knox JJ, Mackenzie MJ (2013). Metastatic non-clear cell renal cell carcinoma treated with targeted agents: characterization of survival outcome and application of the International mRCC Database Consortium criteria. Cancer.

[27] Flanigan RC, Mickisch G, Sylvester R, Tangen C, Van Poppel H, Crawford ED (2004). Cytoreductive nephrectomy in patients with metastatic renal cancer: a combined analysis. J Urol.

[28] Rini BI, Campbell SC (2007). The evolving role of surgery for advanced renal cell carcinoma in the era of molecular targeted therapy. J Urol.

[29] Heng DY, Wells JC, Rini BI, Beuselinck B, Lee JL, Knox JJ (2014). Cytoreductive nephrectomy in patients with synchronous metastases from renal cell carcinoma: results from the International Metastatic Renal Cell Carcinoma Database Consortium. Eur Urol.

[30] Kavolius JP, Mastorakos DP, Pavlovich C, Russo P, Burt Me, Brady MS (1998). Resection of metastatic renal cell carcinoma. J Clin Oncol.

[31] Motzer RJ, Hutson TE, Tomczak P, Michaelson MD, Bukowski RM, Rixe O (2007). Sunitinib versus interferon alfa in metastatic renal-cell carcinoma. N Engl J Med.

[32] Escudier B, Pluzanska A, Koralewski P, Ravaud A, Bracarda S, Szczylik C (2007). Bevacizumab plus interferon alfa-2a for treatment of metastatic renal cell carcinoma: a randomised, double-blind phase III trial. Lancet.

[33] Rini BI, Halabi S, Rosenberg JE, Stadler WM, Vaena DA, Ou SS (2008). Bevacizumab plus interferon alfa compared with interferon alfa monotherapy in patients with metastatic renal cell carcinoma: CALGB 90206. J Clin Oncol.

[34] Sternberg CN, Davis ID, Mardiak J, Szczylik C, Lee E, Wagstaff J (2010). Pazopanib in locally advanced or metastatic renal cell carcinoma: results of a randomized phase III trial. J Clin Oncol.

[35] Motzer RJ, Hutson TE, Cella D, Reeves J, Hawkins R, Guo J (2013). Pazopanib versus sunitinib in metastatic renal-cell carcinoma. N Engl J Med.

[36] Hudes G, Carducci M, Tomczak P, Dutcher J, Figlin R, Kapoor A (2007). Temsirolimus, interferon alfa, or both for advanced renal-cell carcinoma. N Engl J Med.

[37] Motzer RJ, Escudier B, McDermott DF, George S, Hammers HJ, Srinivas S (2015). Nivolumab versus everolimus in advanced renal-cell carcinoma. N Engl J Med.

[38] Choueiri TK, Escudier B, Powles T, Tannir NM, Mainwaring PN, Rini BI (2015). Cabozantinib versus everolimus in advanced renal-cell carcinoma. N Engl J Med.

[39] Motzer RJ, Hutson TE, Glen H, Michaelson MD, Molina A, Eisen T (2015). Lenvatinib, everolimus, and the combination in patients with metastatic renal cell carcinoma: a randomised, phase 2, open-label, multicentre trial. Lancet Oncol.

[40] Rini BI, Escudier B, Tomczak P, Kaprin A, Szczylik C, Hutson TE (2011). Comparative effectiveness of axitinib versus sorafenib in advanced renal cell carcinoma (AXIS): a randomised phase 3 trial. Lancet.

[41] Escudier B, Eisen T, Stadler WM, Szczylik C, Oudard S, Siebels M (2007). Sorafenib in advanced clear-cell renal-cell carcinoma. N Engl J Med.

[42] Motzer RJ, Escudier B, Oudard S, Hutson TE, Porta C, Bracarda S (2008). Efficacy of everolimus in advanced renal cell carcinoma: a double-blind, randomised, placebo-controlled phase III trial. Lancet.

[43] Vera-Badillo F, Templeton A, Duran I, Ocana A, de Gouveia P, Aneja P (2015). Systemic therapy for non-clear cell renal cell carcinomas: a systematic review and meta-analysis. Eur Urol.

[44] Fernández-Pello S, Hoffman F, Tahbaz R, Marconi L, Lam TB, Albiges L (2017). A systematic review and meta-analysis comparing the effectiveness and adverse effects of different systemic treatments for non-clear cell renal cell carcinoma. Eur Urol.

[45] Geynisman DM (2015). Anti-programmed cell death protein 1 (PD-1) antibody nivolumab leads to a dramatic and rapid response in papillary renal cell carcinoma with sarcomatoid and rhabdoid features. Eur Urol.

[46] Tannir N, Jonasch E, Albiges L, Altinmakas E, Ng CS, Matin SF (2016). Everolimus versus sunitinib prospective evaluation in metastatic non clear cell renal carcinoma (ESPN): A randomized multicenter phase 2 trial. Eur Urol.

[47] Armstrong A, Halabi S, Eisen T, Broderick S, Stadler WM, Jones RJ (2016). Everolimus versus sunitinib for patients with metastatic non-clear cell renal cell carcinoma (ASPEN): a multicentre, open-label, randomised phase 2 trial. Lancet Oncol.

[48] Thian Y, Gutzeit A, Koh DM, Fisher R, Lote H, Larkin J (2014). Revised Choi imaging criteria correlate with clinical outcomes in patients with metastatic renal cell carcinoma treated with sunitinib. Radiology.

[49] Seymour L, Bogaerts J, Perrone A, Ford R, Schwartz LH, Mandrekar S (2017). iRECIST: guidelines for response criteria for use in trials testing immunotherapeutics. Lancet Oncol.

[50] Donat SM, Diaz M, Bishoff JT, Coleman JA, Dahm P, Derweesh IH (2013). Follow-up for clinically localized renal neoplasms: AUA guideline. J Urol.

